# Early amplitude-integrated electroencephalography predicts brain injury and neurological outcome in very preterm infants

**DOI:** 10.1038/srep13810

**Published:** 2015-09-08

**Authors:** Juan Song, Falin Xu, Laishuan Wang, Liang Gao, Jiajia Guo, Lei Xia, Yanhua Zhang, Wenhao Zhou, Xiaoyang Wang, Changlian Zhu

**Affiliations:** 1 Department of Neonatology, the Third Affiliated Hospital of Zhengzhou University, Zhengzhou, 450052, China; 2 The Laboratory of Neonatal Brain Injury of Henan Province, Zhengzhou, China; 3 Department of Neonatology, Children’s Hospital of Fudan University, Shanghai, China; 4 Perinatal Center, Sahlgrenska Academy, University of Gothenburg, Gothenburg, Sweden; 5 Center for Brain Repair and Rehabilitation, Institute of Neuroscience and Physiology, University of Gothenburg, Gothenburg, 40503, Sweden

## Abstract

Early amplitude-integrated electroencephalography (aEEG) has been widely used in term infants with brain injury to predict neurodevelopmental outcomes; however, the prognostic value of early aEEG in preterm infants is unclear. We evaluated how well early aEEG could predict brain damage and long-term neurodevelopmental outcomes in very preterm infants compared with brain imaging assessments. We found that severe aEEG abnormalities (p = 0.000) and aEEG total score < 5 (p = 0.006) within 72 h after birth were positively correlated with white-matter damage, but aEEG abnormalities were not associated with intracranial hemorrhage (p = 0.186). Severe abnormalities in aEEG recordings, head ultrasound, and cranial magnetic resonance imaging (MRI) were all positively correlated with poor outcome at 18 months corrected age. The predictive power of poor outcomes of the aEEG and MRI combination was the same as the aEEG, MRI, and head ultrasound combination with a sensitivity of 52.4%, specificity of 96.2%, positive predictive value of 78.6%, and negative predictive value of 88.4%. These results indicate that severely abnormal aEEG recordings within 72 h after birth can predict white-matter damage and long-term poor outcomes in very preterm infants. Thus aEEG can be used as an early marker to monitor very preterm infants.

Preterm birth rates have increased globally since 1990, and about 15 million preterm neonates are currently born every year^1^,^2^,^3^. Modern advances in prenatal and neonatal intensive care have led to an increase in the survival rate of these premature infants^4^. However, neonates born preterm have increased risk of both short-term complications and long-term neurodevelopmental disorders such as cerebral palsy and intellectual disabilities^5^,^6^,^7^, and prematurity is still the leading cause of neonatal death and the second cause of childhood death under the age of 5 years^8^. Quality of life in premature infants who suffer from perinatal brain injury has become a major concern with clear social relevance. Although there are many causes for preterm labor^9^, all brain injuries in preterm infants consist mainly of periventricular leukomalacia (PVL) and intracranial hemorrhage (ICH)^10^.

Early diagnosis and early treatment of brain damage in preterm infants has the potential to improve their long-term quality of life^11^. However, the clinical manifestation of brain damage in preterm infants is not specific and not typical. The majority of ICH occurs in the first 72 hours after birth, especially in the first 24 hours^12^, and PVL occurs later^13^, thus early identification of preterm brain damage through clinical manifestations is difficult. The current clinical method for early diagnosis of brain damage in preterm infants is still mainly through neuroimaging – including head ultrasound (HUS) and magnetic resonance imaging (MRI) – but using neuroimaging for the diagnosis of severe brain damage has some limitations. Evaluation of brain damage, especially white-matter damage (WMD), by head ultrasound usually requires monitoring for more than 2 weeks^14^, and head MRI is difficult to perform in newborn preterm infants who need breathing support. It is necessary, therefore, to explore other methods of early prediction of brain damage in preterm infants.

Amplitude-integrated electroencephalography (aEEG) has the advantages of being simple to perform, of allowing continuous bedside monitoring, and of providing easily interpreted results, all of which make this an important method in the neonatal intensive care unit for monitoring brain function. It has also been shown that aEEG classifications correlate strongly with the clinical degree of hypoxic-ischemic encephalopathy and neurological outcomes in full-term infants^15^. Research on the use of aEEG in preterm infants has shown impressive predictive value for short-term and later outcomes, although such studies have only included limited numbers of patients^16^,^17^,^18^,^19^. The role of aEEG for predicting long-term adverse neurological outcome in preterm infants needs to be further confirmed by comparing it with the results from neuroimaging.

The aim of this study was to evaluate whether aEEG recordings within 72 h after birth predict brain damage and neurodevelopmental outcome in preterm infants with gestational age (GA) less than 32 weeks, and to compare these outcomes to head ultrasound and MRI assessments.

## Results

### Baseline characteristics

**Figure 1:**
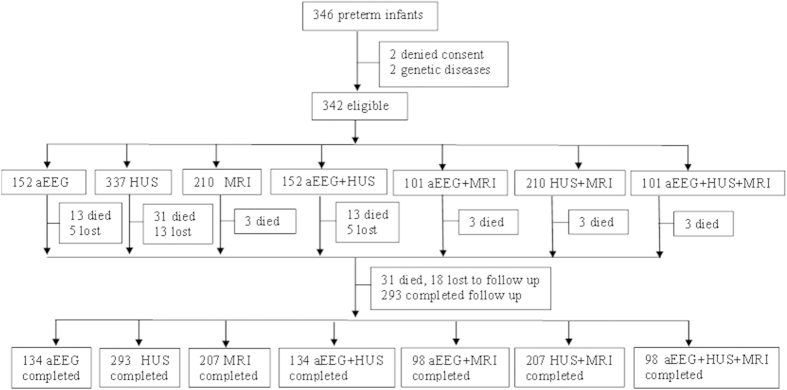
Study flow. A schematic flowchart describing the recruitment and neurodevelopmental follow-up evaluation from birth to 18 months of corrected age for the preterm infants. Lost to follow-up means that contact with the family was lost during the follow-up period. aEEG: amplitude-integrated electroencephalography; HUS: head ultrasonography; MRI: magnetic resonance imaging.

A total of 346 infants were admitted to the NICU during the study period. Four infants were excluded based on the exclusion criteria and 342 were eligible, of which 18 were lost to follow-up and excluded from the analysis. A total of 324 preterm infants with an average GA of 30.0 ± 1.1 weeks (27.1–31.6 weeks) and average birth weight of 1377 ± 288 g (700–2200 g) were included (Table 1). The male to female ratio was 1.8:1. A total of 152 very preterm infants underwent aEEG within 72 h, 337 preterm infants underwent head ultrasonography, 210 preterm infants underwent MRI at 40 weeks of corrected age, 152 preterm infants underwent both aEEG and head ultrasonography, 101 preterm infants underwent both aEEG and MRI, 210 preterm infants underwent both head ultrasonography and MRI, and 101 preterm infants underwent all three examinations. There were 31 preterm infants who died at an average age of 15.2 ± 12.5 days, of which 15 died from brain damage and 16 died from other causes including respiratory failure (n = 7), sepsis (n = 6), perforation of the digestive tract (n = 1), or pulmonary hemorrhage (n = 2), and these were excluded from the analysis of neurodevelopmental outcomes. A total of 308 infants were considered for long-term analysis, but 18 were lost to follow-up. Excluding those lost to follow-up, there were 134 follow-ups for infants who underwent aEEG within 72 h (88.15%), 293 follow-ups for infants who underwent head ultrasonography (86.94%), and 207 follow-ups for infants who underwent brain MRI at 40 weeks of corrected age (98.57%) (Fig. 1).

### aEEG recordings and brain damage in preterm infants

There was a significant association between the classification of aEEG recordings within 72 h after birth and the degree of WMD in preterm infants. Although aEEG recordings were severely abnormal in 77.8% (7/9) of the infants with grade III or IV ICH, the degree of abnormality of the aEEG recordings in the first 72 h of life were not associated with ICH classification (Table 2). Logistic regression analysis showed that severe aEEG abnormality was positively correlated with WMD (adjusted regression coefficients: 1.66; S.E.: 0.40; p = 0.000; OR: 5.28; 95% CI [2.41–11.57]). This means that severe aEEG abnormalities could predict WMD. Further analysis with the aEEG scoring system showed that cycling score, narrow bandwidth score, and total score on aEEG within 72 h in infants with severe WMD were significantly lower than the scores of those without or with mild WMD (Table 3). Logistic regression analyses showed that an aEEG total score < 5 positively correlated with WMD (adjusted regression coefficients: 1.39; S.E.: 0.50; p = 0.006; OR: 4.01; 95% CI [1.50–10.68]). However, ICH was not associated with aEEG scores. This indicates that an aEEG total score < 5 could predict WMD.

### aEEG recordings and poor outcome

Of the 152 preterm infants who underwent aEEG within 72 h after birth, 5 were lost to follow-up, 13 died (5 of them from brain damage), 134 surviving infants were followed up to 18 months of age, and 139 infants (including 5 that died from brain damage) were included in the statistical analysis of poor outcome. Abnormal aEEG recordings within 72 h were significantly associated with poor outcomes, including cerebral palsy and hypophrenia in preterm infants at 18 months corrected age (Table 4).

### Head ultrasound and poor outcome

Of 337 infants who underwent head ultrasound within 1 month after birth, 13 were lost to follow-up, 31 died (15 from brain damage), 293 surviving infants were followed up to 18 months of corrected age, and 308 infants were included in the statistical analysis of poor outcome. Abnormalities in the head ultrasound within 1 month were statistically associated with poor outcomes, including cerebral palsy, hypophrenia, and death from brain injury in preterm infants at 18 months corrected age (Table 4).

### Head MRI and poor outcome

Of 210 infants who underwent head MRI at 40 weeks corrected age, 3 infants died from brain damage, 207 surviving infants were followed up to 18 months of age, and 210 infants were included in the statistical analysis of poor outcome. Abnormality of head MRI at 40 weeks corrected age was statistically associated with poor outcomes, including cerebral palsy, hypophrenia, audio-visual disorder, and death in preterm infants at 18 months corrected age (Table 4).

### Combined examinations and poor outcomes

The combined examinations and long-term outcomes were analyzed for different combinations of assessments (Table 5). Of 101 preterm infants who underwent all three examinations, 3 infants died (2 from brain damage), 98 surviving infants were followed up to 18 months of age, and 100 infants were included in the statistical analysis of poor outcomes. Results from preterm infants who underwent both aEEG and MRI were the same as those who underwent all three examinations. Of 152 preterm infants who underwent both aEEG and head ultrasound, 5 were lost to follow-up, 13 infants died (5 from brain damage), 134 surviving infants were followed up to 18 months of age, and 139 infants were included in the statistical analysis of poor outcomes. Of 210 preterm infants who underwent both head ultrasound and MRI, 3 infants died (2 from brain damage), 207 surviving infants were followed up to 18 months of age, and 209 infants were included in the statistical analysis of poor outcomes. Abnormalities in the combinations of two or three examinations were statistically associated with poor outcomes, including cerebral palsy and hypophrenia in preterm infants at 18 months corrected age (Table 5).

### Predictors of poor outcomes

Univariate analysis of poor outcome was performed considering 22 items, including GA, birth weight, severe asphyxia, mechanical ventilation >7 days, respiratory distress syndrome grade 3 or 4, severe anemia, persistent hypoxemia, persistent hypercapnia, persistent hypoglycemia, sepsis, bronchopulmonary dysplasia, necrotizing enterocolitis, cholestasis, family socioeconomic status, parent education level, severe abnormality in aEEG, HUS, MRI, aEEG + HUS, aEEG + MRI, HUS + MRI, and aEEG + HUS + MRI. All combinations of examinations were analyzed separately, and p-values < 0.05 were required for inclusion in the logistic regression analysis. Logistic regression analyses showed that severe aEEG abnormality (adjusted regression coefficients: 1.61; S.E.: 0.63; p = 0.011, OR: 5.00; 95% CI [1.45–17.22]), severe HUS abnormality (adjusted regression coefficients: 1.03; S.E.: 0.41; p = 0.011, OR: 2.80; 95% CI [1.26–6.20]), and severe MRI abnormality (adjusted regression coefficients: 2.82; S.E.: 0.69; p = 0.000, OR: 16.75; 95% CI [4.35–64.50]) were significantly high risk factors of poor outcome. Severe abnormality of aEEG + MRI was a significantly high risk factor of poor outcome (adjusted regression coefficients: 3.92; S.E.: 0.96; p = 0.000, OR: 50.54; 95% CI [7.72–331.09]), which was the same as aEEG + HUS + MRI. Severe abnormalities of aEEG + HUS (adjusted regression coefficients: 2.09; S.E.: 0.63; P = 0.001, OR: 8.05; 95% CI [2.34–27.71]) and HUS + MRI (adjusted regression coefficients: 3.36; S.E.: 0.75; p = 0.000, OR: 28.75; 95% CI [6.68–123.76]) were also positively correlated with poor outcome. We took severe abnormalities of different combinations as predictors, and there was no significant predictive difference among different combinations of the assessments (Table 5). The sensitivity, specificity, positive predictive value, and negative predictive value for predicting poor outcomes and cerebral palsy for all combinations of assessments are shown in Table 6.

## Discussion

This study used a relatively large sample to show that severe abnormalities of early aEEG can predict white-matter injury and long-term outcomes in preterm infants. The specificity of aEEG to predict poor outcomes was similar to head ultrasound but lower than cranial MRI, and the sensitivity of aEEG was highest compared to MRI and HUS.

The survival of premature infants has improved dramatically due to advances in neonatal intensive care. However, preterm infants are at increased risk of dangerous complications occurring during the neonatal period that often cause brain injury such as ICH and PVL, and these are the most important risk factors for long-term neurologic sequelae such as cerebral palsy, cognitive deficits, and learning impairments^20^. Neuroimaging has played an important role in the diagnostic evaluation and monitoring of preterm infants^10^, but even though HUS and cranial MRI have predictive value for long-term outcomes there remains a subset of infants with impairments in childhood who demonstrate no significant brain injury or alterations upon neuroimaging^21^. Neural dysfunction as reflected by aberrations in aEEG has been widely used as an early marker of brain injury in full-term infants suffering from asphyxia, but the use of aEEG in preterm infants is not very common. Recent studies have found that abnormal aEEG in preterm infants is associated with brain injury and later outcome^16^,^22^,^23^, and the use of aEEG at earlier time points after birth might open a potential time window for neuroprotective interventions.

The optimal time after birth for taking aEEG recordings to predict adverse outcomes is still unclear^17^,^18^,^24^. Recent studies have assessed aEEG recordings within 72 h after birth and found reasonable predictive value for these aEEG recordings in predicting poor outcomes^18^,^22^,^25^. Preterm infants are most vulnerable to high-risk perinatal factors within the first 72 h after birth, and most ICH in preterm infants occurs during this period of time^12^. In addition, abnormalities in aEEG recordings taken during this time can predict hospitalization time in preterm infants^26^. In this study, we used aEEG recordings taken within the first 72 h of life to evaluate the predictive value of abnormal aEEG compared to standard imaging assessments. Univariate analysis showed that the degree of abnormality in the aEEG recordings in the first 72 h of life was positively associated with the degree of WMD. Multivariate analysis showed that severe aEEG abnormality was a significant higher risk of WMD and can be taken as an early predictor of WMD in preterm infants. We also found that cycling score, narrow bandwidth score, and total score on aEEG within 72 h in infants with severe WMD were significantly lower than in infants with no or mild WMD. This indicates that preterm infants with severe WMD have a delay in brain maturation^27^. Kato T^28^ reported 1 case of preterm infant developed PVL with GA 29 weeks, whose aEEG recordings in the first 1 hour was at the inhibitory state. In this study, we found that an aEEG total score <5 was a significant risk for WMD. These early maturity changes in aEEG recordings can help predict the occurrence of WMD in preterm infants.

The abnormal aEEG recordings had no association with the classification of ICH. This means that a preterm infant with severely abnormal aEEG recordings soon after birth will not necessarily develop severe ICH, which is different from other reports showing that early aEEG recordings are associated with ICH^17^,^29^. One possible reason for this is that aEEG records the activity of the brain cortex and reflects activity from the underlying cortex such as WMD. Although grade IV ICH might be reflexed by aEEG, there was only one patient with grade IV ICH in our study, and the influence of grade I–III ICH on the electrophysiological activity of the brain cortex might be less. Another reason for this might be that there were only nine very preterm infants with severe ICH (grade III–IV) in our study, which might affect the statistical results. Further study with a larger sample is necessary to confirm these results.

The predictive value of early aEEG for long-term outcomes is still controversial. Recent studies^22^,^30^ reported that abnormal aEEG recordings within 24 hours after birth in infants with GA 22–30 weeks or 27–32 weeks were associated with long-term adverse neurodevelopmental outcomes. However, another study reported that aEEG recordings in preterm infants with GA 28–36 weeks could not predict outcome at 18 to 22 months of age^31^. In the current study, we found that severe abnormalities in aEEG recordings within 72 h after birth were positively correlated with poor outcomes at 18 months of corrected age in preterm infants less than 32 weeks GA. The contradictory predictive values could be related to different GA of the selected preterm infants because EEG activity is related to brain maturation.

Neuroimaging of preterm infants has become part of routine clinical care in the NICU, and HUS is still considered the method of first choice. However, sequential HUSs are required to evaluate preterm brain injury and to predict outcome. The sensitivity and specificity of HUS in preterm infants ranges from 45% to 90%^16^. In this study, the sensitivity and specificity of HUS for cerebral palsy were 87.5% and 64.5%, respectively, which were a little bit higher than the assessment by aEEG. MRI is increasingly used for preterm infants to detect brain injury and to evaluate long-term outcomes, but it can only be performed when the infant has stabilized or is at term equivalent age^10^. MRI has been reported to have high predictive value with a sensitivity of 84% and specificity of 89% for preterm infants when performed at term equivalent age^32^. In our cohort, the specificity was similar to the previous report, but with a lower sensitivity for predicting poor outcomes or cerebral palsy. The predictive value of combining aEEG and cerebral MRI was the same as the combination of all three assessments with the highest sensitivity (83.3%) and specificity (96.6%) as well as positive predictive value (76.9%) and negative predictive value (97.7%) for cerebral palsy in preterm infants.

In summary, severe abnormalities in aEEG recordings within 72 h after birth could predict WMD in preterm infants with GA less than 32 weeks. Severe abnormalities in aEEG recordings, HUS, and cranial MRI were all positively correlated with poor outcome at 18 months corrected age. The predictive value of combined aEEG and MRI for poor outcome in preterm infants less than 32 weeks is similar to all three examinations combined together. We conclude that aEEG has the potential for use as an early marker to monitor preterm infants in the NICU.

## Patients and Methods

### Subjects

Neonates with GA < 32 weeks admitted to the NICU within 72 h after birth at the Third Affiliated Hospital of Zhengzhou University, China, from January to December 2012 were screened for eligibility for this prospective study cohort. Neonates with congenital brain malformations, chromosomal diseases, genetic diseases, or metabolic diseases were excluded. This study was approved by the Human Research Ethics Committee and Clinical Trials Committee of the hospital in accordance with the Helsinki Declaration. Written informed parental consent was obtained for all neonates.

### aEEG

aEEG traces were recorded within 72 h (43 infants within 24 h, 67 infants between 24 h and 48 h, and 37 infants between 48 h and 72 h) after birth using a NicoletOne^TM^ device (Nicolet Biomedical Inc., Madison, WI, US). Each recording lasted for 4–24 hours (91 infants were recorded for 4–10 h, 35 infants were recorded for 10–16 h, and 21 infants were recorded for 16–24 h). The average recording time was 10.9 ± 5.5 h, and all aEEG recordings were obtained by two investigators. The aEEG recordings were assessed using a combination of criteria for amplitude, background activity, and sleep-wake cycling (SWC): (1) normal: continuous normal amplitude (upper margin >10 μV and lower margin >5 μV), SWC matched to corresponding age, and no electrographic seizures; (2) mildly abnormal: discontinuous activity and mildly abnormal amplitude (upper margin >10 μV and lower margin <5 μV) with immature and delayed SWC or normal amplitude with electrographic seizures; (3) severely abnormal: discontinuous activity and severely abnormal amplitude (upper margin <10 μV and lower margin <5 μV) without SWC, including burst-suppression (discontinuous activity with lower margin at 0–1 μV constantly and a burst amplitude >25 μV), flat trace (electrical silence), continuous low voltage (continuous very low amplitude activity at about 5 μV or below 5 μV), or mildly abnormal amplitude with electrographic seizures^33^,^34^. aEEG scores were assigned according to a scoring system described by Burdjalov *et al.* to assess brain maturity^35^. The scores include four individual scores for continuity, cycling, amplitude, and bandwidth of the lower border, which can be summed into a total score ranging from 0 to 13 with higher scores being correlated to increasing postconceptional age.

### Head ultrasonography

HUS was performed within 3 days after birth and then weekly until 4 weeks after birth with a GE^TM^ Voluson (General Electric Company, Fairfield, CT, US) with a 7.5-MHz transducer. ICH was classified as grade I to IV according to Papile *et al.*^36^. The WMD was classified into the following three types according to de Vries^37^: (1) no WMD (no enhancement of the echo of cerebral white matter); (2) mild WMD (transient and slight enhancement of the echo within 3 days after birth but decreasing or disappearing after 7–10 days); and (3) severe WMD (significant enhancement of the echo and PVL or a decrease in cerebral white-matter volume in the primary lesion after 3–4 weeks). Severe abnormal HUS was defined in this study as grade III or IV ICH or severe WMD.

### Head MRI

Head MRI was performed at 40 weeks corrected age using a 1.5 T MRI (General Electric Company). Abnormalities on MRI at term equivalent were assessed blindly by the scores of white matter and gray matter^38^. Severely abnormal MRI was defined in this study as PVL or severe dilatation of the lateral ventricles.

### Follow-up

All of the preterm infants were tracked every month for 6 months and then every 3 months until 18 months corrected age. All of the surviving preterm infants were evaluated through gross neurologic assessment and mental developmental index (MDI) testing. Assessment of neuromotor disability was based on the presence of cerebral palsy and functional disability. Mental or psychological development was evaluated with the Bayley Scales of Infant Development, Second Edition^39^. Good outcome was defined as survival without neurodevelopmental impairment, which includes one or more of the following: cerebral palsy, hypophrenia (MDI < 70, Bayley scales), or audio-visual disorder. Poor outcome was defined as survival with one or more of cerebral palsy, hypophrenia, or audio-visual disorder or death due to brain damage.

### Statistical analysis

Statistical analysis was performed using SPSS version 19.0. Quantitative data are presented as means ± standard deviation. Differences between groups were evaluated using one-way ANOVA and LSD *t*-test. A *p*-value < 0.05 was considered significant. Qualitative data were compared using the chi-square tests, and a *p*-value < 0.05 suggested an association between the two groups. Logistic regression analyses were used to assess risk factors of WMD and poor outcome. Sensitivity, specificity, positive predictive value, and negative predictive value were calculated using diagnostic tests.

## Additional Information

**How to cite this article**: Song, J. *et al.* Early amplitude-integrated electroencephalography predicts brain injury and neurological outcome in very preterm infants. *Sci. Rep.* **5**, 13810; doi: 10.1038/srep13810 (2015).

## Figures and Tables

**Table 1:** Epidemiological data of the study group.

	All infants (n = 324)	Favorable outcome (n = 262)	Poor outcome (n = 62)
Gestational age (weeks)	30.0 ± 1.1	30.2 ± 1.0	29.5 ± 1.2*
Birth weight (g)	1377 ± 288	1562 ± 198	1093 ± 131*
Male sex, n (%)	208 (64.2)	167 (63.7)	41 (66.1)
1-minute Apgar < 3′, n (%)	16 (4.9)	10 (3.8)	6 (9.7)*
1-minute Apgar 3–7′, n (%)	152 (46.9)	104 (39.7)	48 (77.4)*
1-minute Apgar > 7′, n (%)	156 (48.2)	148 (56.5)	8 (12.9)*
Mechanical ventilation, n (%)	126 (38.9)	75 (28.6)	51 (82.3)*
Sepsis, n (%)	58 (17.9)	32 (12.2)	26 (41.9)*
BPD, n (%)	38 (11.7)	26 (9.9)	12 (19.4)*
NEC, n (%)	11 (3.4)	8 (3)	3 (4.8)
Severe anemia, n (%)	118 (36.4)	88 (33.6)	30 (48.4)*
Cholestatic syndrome, n (%)	20 (6.2)	15 (5.7)	5 (8.1)
Persistent hypoxemia, n (%)	96 (29.6)	68 (26.0)	28 (45.2)*
Persistent hypercapnia, n (%)	30 (9.3)	17 (6.5)	13 (21.0)*
Persistent hypoglycemia, n (%)	7 (2.2)	6 (2.3)	1 (1.6)
Parent’s monthly income			
<3000 yuan, n (%)	42 (13.0)	30 (11.5)	12 (19.4)
3000–8000 yuan, n (%)	201 (62.0)	161 (61.5)	40 (64.5)
>8000 yuan, n (%)	81 (25.0)	71 (27.0)	10 (16.1)
Parent’s education level			
Middle school, n (%)	45 (13.9)	34 (13.0)	11 (17.7) *
High school, n (%)	172 (53.1)	132 (50.4)	40 (64.5) *
University, n (%)	107 (33.0)	96 (36.6)	11 (17.7) *

BPD: bronchopulmonary dysplasia. NEC: necrotizing enterocolitis. Poor outcome: death or survival with cerebral palsy, hypophrenia, or audio-visual disorder. Favorable outcome: survival without cerebral palsy, hypophrenia, or audio-visual disorder. *P < 0.05 significant difference between infants with favorable outcome versus poor outcome at 18 months corrected age.

**Table 2:** aEEG classification compared to brain damage severity in preterm infants.

	WMD			ICH		
	No WMD	Mild	Severe	No ICH	ICH I-II	ICH III-IV
Normal aEEG (n = 10)	6	1	3	9	1	0
Mildly abnormal aEEG (n = 70)	13	38	19	36	32	2
Severely abnormal aEEG (n = 67)	6	19	42	31	29	7
P-value	**0.000**			0.186		

WMD: white-matter damage. ICH: intracranial hemorrhage. P-value: assessed the association between the classification of aEEG with the degree of WMD and ICH using chi-square test, and P < 0.05 was considered significant.

**Table 3:** Relationship between 72 h aEEG scores and brain damage in preterm infants.

	No WMD n = 25	Mild WMD n = 58	Severe WMD n = 64	P-value	No ICH n = 76	ICH I-IIn = 62	ICH III-IV n = 9	P-value
Co	1.95 ± 0.41	1.80 ± 0.58	1.64 ± 0.59	0.105	1.75 ± 0.58	1.78 ± 0.56	1.50 ± 0.55	0.535
Cy	1.81 ± 1.44	1.77 ± 1.50	0.77 ± 1.25*	**0.006**	1.22 ± 1.40	1.24 ± 1.46	1.17 ± 1.84	0.920
LB	1.21 ± 0.71	1.14 ± 0.60	0.96 ± 0.62	0.233	1.09 ± 0.75	1.04 ± 0.48	1.00 ± 0.63	0.910
B	1.68 ± 0.82	1.59 ± 0.89	1.23 ± 0.58*	**0.022**	1.48 ± 0.79	1.36 ± 0.75	1.33 ± 0.52	0.709
T	6.05 ± 2.48	6.15 ± 2.89	4.60 ± 2.34*	**0.012**	5.45 ± 2.85	5.32 ± 2.37	4.83 ± 2.92	0.859

Co: continuity score. Cy: sleep-wake cycling score. LB: lower border amplitude score. B: narrow bandwidth score. T: total score. WMD: white matter damage. ICH: intracranial hemorrhage. P-value: compared aEEG scores with WMD and ICH using one-way ANOVA. *P < 0.05, compared with no and mild WMD using LSD-*t* test.

**Table 4:** aEEG, HUS, and MRI examination and poor outcome at 18 months in preterm infants.

	Poor outcome	CP	Hypophrenia	Audio-visual disorder	Death
aEEG					
Normal (n = 9)	0	0	0	0	0
Mildly abnormal (n = 67)	6	2	4	1	2
Severely abnormal (n = 63)	18	10	14	1	3
P-value	**0.005**	**0.018**	**0.009**	0.928	0.721
HUS					
Normal (n = 46)	1	1	1	0	0
Mildly abnormal (n = 144)	15	1	8	5	3
Severely abnormal (n = 118)	30	14	16	1	12
P-value	**0.013**	**0.000**	**0.008**	0.203	**0.003**
MRI					
Normal (n = 148)	11	2	10	1	0
Mildly abnormal (n = 32)	5	3	4	1	0
Severely abnormal (n = 30)	13	11	10	3	3
P-value	**0.000**	**0.000**	**0.000**	**0.005**	**0.000**

CP: cerebral palsy, aEEG: amplitude-integrated electroencephalography, HUS: head ultrasound, MRI: cranial magnetic resonance imaging. Hypophrenia: MDI < 70 according to Bayley Scales. Death: patients died from brain damage. Poor outcome: patients died from brain damage or survived with one or more of CP, hypophrenia, or audio-visual disorder. P-value: assessed the association between classifications of aEEG/HUS/MRI with poor outcomes using chi-square test, and P < 0.05 was considered significant.

**Table 5:** Prediction of poor outcome by combined aEEG, HUS, and MRI in preterm infants.

Combined examinations (n)	Poor outcome % (n/total)	CP % (n/total)	Hypophrenia % (n/total)	Audio-visual Disorder n/total	Death n/total
HUS + MRI (209)	45.2% (14/31)*	68.8% (11/16)*	41.7% (10/24)*	2/5	2/2
aEEG + HUS (139)	66.7% (16/24)*	83.3% (10/12)*	66.7% (12/18)*	1/2	3/5
aEEG + MRI (100)	52.4% (11/21)*	83.3% (10/12)*	50% (9/18)*	1/2	1/2
aEEG + HUS + MRI (100)	52.4% (11/21)*	83.3% (10/12)*	50% (9/18)*	1/2	1/2
P-value	0.159	0.098	0.303	0.999	0.619

HUS + MRI, aEEG + HUS, aEEG + MRI, aEEG + HUS + MRI: infants undergoing different combinations of examinations. CP: cerebral palsy, aEEG: amplitude-integrated electroencephalography, HUS: head ultrasound, MRI: cranial magnetic resonance imaging. Hypophrenia: MDI < 70 according to Bayley Scales. Death: patients died from brain damage. Poor outcome: patients died from brain damage or survived with one or more of CP, hypophrenia, or audio-visual disorder. n/total: patients with severe abnormalities of different combinations/all patients. P-value: compared the assessments of poor outcomes with severe abnormalities of different combinations using chi-square tests. *P < 0.05 compared abnormalities of different combinations with poor outcomes using chi-square test.

**Table 6:** Prediction of CP or poor outcome at 18 months corrected age in preterm infants.

Predictor	CP				Poor outcomes			
	Sensitivity (%)	Specificity (%)	PPV (%)	NPV (%)	Sensitivity (%)	Specificity (%)	PPV (%)	NPV (%)
aEEG (n = 139)	83.3	58.2	15.9	97.4	75.0	60.9	28.6	92.1
HUS (n = 308)	87.5	64.5	11.9	98.9	65.2	66.4	25.4	89.7
MRI (n = 210)	68.8	90.2	36.7	97.2	50.0	92.1	53.3	91.1
aEEG + HUS (n = 139)	83.3	78.7	27.0	98.0	66.7	79.1	40.0	91.9
aEEG + MRI (n = 100)	83.3	96.6	**76.9**	**97.7**	52.4	96.2	**78.6**	**88.4**
HUS + MRI (n = 209)	68.8	93.8	47.8	97.3	45.2	93.8	56.0	90.8
aEEG + HUS + MRI (n = 100)	83.3	96.6	**76.9**	**97.7**	52.4	96.2	**78.6**	**88.4**

The predictors are defined as severe abnormalities in aEEG recordings within 72 h, HUS, or MRI individually or in combination. CP: cerebral palsy, aEEG: amplitude-integrated electroencephalography HUS: head ultrasound, MRI: cranial magnetic resonance imaging, PPV: positive predictive value, NPV: negative predictive value. Poor outcome: patients died from brain damage or survived with one or more of CP, hypophrenia, or audio-visual disorder.
